# The Role of Natriuretic Peptides for the Diagnosis of Left Ventricular Dysfunction

**DOI:** 10.1155/2013/784670

**Published:** 2013-09-28

**Authors:** Alberto Palazzuoli, Matteo Beltrami, Gaetano Ruocco, Marco Pellegrini, Ranuccio Nuti

**Affiliations:** Department of Internal Medicine and Metabolic Diseases, Cardiology Section, Le Scotte Hospital, 53100 Siena, Italy

## Abstract

Natriuretic peptides (NPs) are entered in current guidelines for heart failure (HF) diagnosis and management because of their high specificity and sensibility in screening patients with acute dyspnea. Due to their availability and relatively low cost, they became the first step examinations in HF patients evaluation at hospital admission together with clinical and chest radiography examination. NPs are released following any cardiac haemodynamic stress due to volume or pressure overload and should be considered as a mirror of cardiac condition helping in recognizing patients with poor outcome. Moreover, the exact role of NPs in early HF stages, in isolated diastolic dysfunction, and in general population is questioned. Several promising reports described their potential role; however, the wide cut-off definition, inclusion criteria, and intrinsic measurement biases do not actually consent to their clinical application in these settings. A multimodality strategy including both NPs and imaging studies appears to be the best strategy to define the cardiac dysfunction etiology and its severity as well as to identify patients with higher risk. In this review, we describe the current and potential role of NPs in patients with asymptomatic cardiac insufficiency, evaluating the requirement to obtain a better standardization for imaging as for laboratory criteria.

## 1. Introduction

Research regarding the role of serum biomarkers in patients with cardiovascular diseases has grown exponentially over the last ten years. In particular, multiple novel biomarkers have been developed for heart failure (HF), due in part to a well-established pathophysiology, including cardiac dysfunction, neurohormonal activation, and hydrosaline retention [1–3]. At the same time, the diversity of HF biomarker development reflects the heterogeneity of patients, with multiple etiologies, phenotypes, and comorbidities. In general, four categories of HF biomarkers have been developed, and these biomarkers should reflect the pathophysiology and heterogeneity of HF. The 4 categories are biomarkers (1) of cardiac injury, (2) adrenergic overdrive, (3) inflammation, (4) oxidative stress, and (5) systemic organ damage [4].

Although the classification of biomarkers helps differentiate each type, this also highlights a potential shortcoming of biomarker development: the fact that they are often studied in isolation, though this trend is recently changing. The ideal laboratory tool fulfills the three criteria outlined by Morrow and de Lemos. Traditionally, three different groups of biomarkers have been identified: (1) laboratory tests, such as natriuretic peptides (NPs) and troponins are defined as “laboratory or molecular biomarkers,” (2) those related to signal, imaging, and functional tests are defined as “functional biomarkers,” and (3) those related to genetic polymorphisms and other genomic tests are defined as “genetic biomarkers.” In this sense, an integrate approach appears mandatory to establish early diagnosis and to optimize the outcome. Moreover, a cardiovascular biomarker can be classified in multiple categories according to its pathophysiological characteristics and/or clinical use. Although this classification is arbitrary, since each index can contain several of these characteristics, it is useful for interpreting the value of an individual clinical biomarker. 

Regarding laboratory biomarkers, Morrow and de Lemos outlined the three major criteria of a clinically useful biomarker: (1) accurate, reproducible measurements must be rapidly available to the clinician at a reasonable cost; (2) the biomarker must provide additional information beyond careful clinical assessment; (3) knowledge of the measured level should aid in medical decision making [5]. In clinical practice, it is hard to find a specific biomarker that contains all these characteristics. For example, significant troponin increase in patients with chest pain and any specific ST wave alterations address physician towards an ischemic etiology; similar model should be applied in patients with dyspnea in the absence of specific chest radiography or ECG signs whereby an increase in NPs levels leads to HF diagnosis [4, 6]. 

In the last decade, an “explosion” of data regarding the use of biomarkers in cardiovascular diseases is evident in the literature. This has happened for several reasons: to facilitate diagnosis, improve prognosis both early and late in the course of the disease, and guide management. However, each biomarker has various strengths and weaknesses: some requirements are linked to chemical detection (reproducibility and accuracy, in vivo and in vitro stability, low cost, sensitivity and availability, and international standardization reference tested for gender and age), while others need to respond to clinical requirements (good diagnostic and prognostic accuracy, useful in therapy monitoring, and reasonable cost-effectiveness ratio) [5, 7].

Laboratory tests are able to identify serial mechanisms potentially involved in the genesis of HF; however, it appears hard to find a single specific biomarker including diagnostic, prognostic, and clinical information all together at the same time. In this context, it appears difficult to identify through laboratory tests the early mechanisms that cause myocardial damage, in order to recognize those subjects at risk to develop the HF syndrome. Frequently, a single biomarker demonstrates exciting results in preliminary studies that cannot be confirmed later in prospective multicenter studies [6].

Early diagnosis of HF depends on the availability of specific, accurate, and effective markers of the disease. For this reason, a multimarker strategy including new and old biomarkers together with traditional diagnostic tools has been recently proposed. 

Recently, Braunwald classified biomarkers on the basis of each pathophysiologic process that is involved in HF [7]. This classification is now universally accepted: inflammation, neurohormones, myocyte Injury, Oxidative Stress, and extracellular matrix remodelling biomarkers (Table 1). 

In the HF setting, patients present with various clinical presentations and in distinct stages of progression; therefore, an ideal biomarker should be able to recognize not only disease severity but also the underlying causal mechanisms and the risk profile [8]. Moreover, application in clinical practice requires validation of each biomarker in multiple studies with a unique cutoff, given the categorical nature of the physician decision making. Ideally, this would include studies yielding diagnostic, prognostic, and therapeutic values further validated in the “real-world” setting for example, the evidence that triage guided by biomarkers improves the quality of treatment and shortens the diagnostic process. Probably an ideal biomarker that is able to include all this information is far from being conceived. Perhaps a multimarker strategy as applied in the acute coronary syndrome could be useful to better understand the etiology of HF and its severity and to assess early as well as long term risks. In this context, significant troponin increase associated with a high level of NPs is able to identify patients with myocardial infarction and the poor outcome for HF development [9, 10].

## 2. Haemodynamic and Nonhaemodynamic Determinants in Acute Heart Failure

Multiple pathophysiologic factors contribute to the development of HF and cardiac impairment. For simplicity, three principal disorders predominate: pump dysfunction, neurohormonal activation, and salt-water retention. However, the clinical presentation resulting from these underlying disorders varies greatly, in part determined by hemodynamic status, primary cardiac disorder, systemic pressure, and organ perfusion/damage [11, 12]. A recent report has divided the acute decompensated HF syndromes on the basis of primary cardiac dysfunction, coronary artery disease presence, and clinical presentation [1, 10]. Further subclassification by type of cardiac dysfunction has been proposed: most patients with HF have both systolic and diastolic left ventricular (LV) dysfunction, but in some cases the syndrome can occur with isolated systolic or diastolic dysfunction. HF with preserved left ventricular ejection fraction (HFpEF) is characterized by a nondilated, usually hypertrophied, left ventricle in which left ventricular ejection fraction (LVEF) is preserved at rest, and the parameters of LV relaxation and filling are markedly deranged. Patients with HFpEF are a heterogeneous and understudied group that includes subjects with both hypertensive heart disease and hypertrophic cardiomyopathy [11]. 

**Figure 1:**
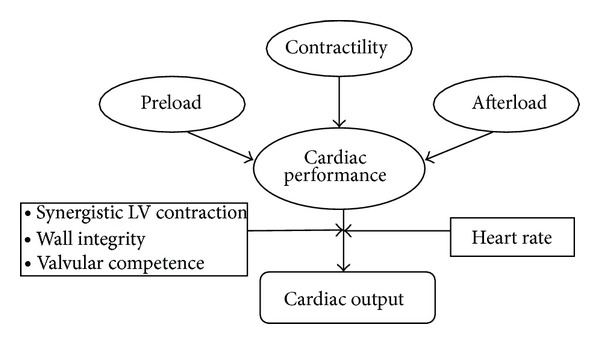
Haemodynamic and intrinsic cardiac factors influencing filling pressure and cardiac performance.

However, systolic HF is itself a heterogeneous condition with several mechanisms acting as potential contributors: preload, afterload, cardiac contractility and filling, peripheral vascular resistance, and heart rate variability are all important determinants of cardiac performance. The presence of viable myocardium, extension of necrosis, and severity of coronary disease are further determinants of LV function. One of the determinants is the diastolic function that is intimately related to cardiac afterload, and this load dependence is amplified in HF [12]. Acute increases in afterload lead to prolongation of isovolumic relaxation. Loading increases in systolic pressure have further impairing effects on the diastole. This increased passive stiffness, together with abnormal relaxation, will shift the diastolic pressure-volume relation curve upward and to the left, with retrograde elevation of LV end-diastolic, left atrial, and pulmonary pressures. Reduced myocardial release, augmented myocardial mass and stiffness, and delayed inactivation are all elements that contribute significantly to an increase in LV filling pressure and wall tension [13]. Beyond the hemodynamic factors, there are at least two principal actors playing an equally influential role in HF establishment and maintenance: neurohormonal activation and hydrosaline retention. Both mechanisms cause peripheral vasoconstriction that results in an increased LV afterload, the activation of inflammatory pathways, the increase of growth factors and endothelial dysfunction, and the induction of oxidative stress. The increased fluid overload results in elevated intracardiac pressure as well as pulmonary congestion [14]. It would be difficult to obtain all this information by a single biomarker; therefore, a multimarker strategy including clinical laboratory and imaging parameters is currently the most appropriate strategy. Among all the potential biomarkers, the ones that probably contain the most clinical hemodynamic and systemic information are NPs. Such dowries are due to its secretion mechanism: NPs are increased as a consequence of augmented cardiac pressure, volume overload and wall tension. NPs levels are also mediated by means of the renin-angiotensin system and neuroendocrine overdrive; therefore, for specific counter regulatory mechanisms, levels are augmented during idrosaline status retention. For all these reasons, NPs are able to provide some additive and complementary information with respect to the traditional tools in the HF setting [15] (Figure 1).

## 3. The Current and Potential Role of NP in Heart Failure

The discovery of NPs as diagnostic biomarkers has been one of the most important advances in the diagnosis of heart failure. Prior to NPs, several studies suggested the need to improve diagnosis, especially with the increasing prevalence of heart failure in the United States [26]. However, diagnosis may be delayed, due to the aspecificity of symptoms and the previous lack of a rapid, easily accessible, bedside gold standard protocol to facilitate diagnosis. 

### 3.1. NP in Emergency Setting

The utility of NPs was initially demonstrated in emergency department (ED) patients presenting with acute dyspnea. B-type natriuretic peptide (BNP) levels were a more accurate predictor of HF diagnosis than history, physical exam and routine laboratory tests [16]. The Breathing Not Properly trial showed that a BNP level ≤ 100 pg/mL yielded 90% sensitivity and 76% specificity in separating cardiac from noncardiac etiologies of dyspnea [17]. N-terminal pro-B-type natriuretic peptide (NT-proBNP) has also been studied in the ED, during hospitalization and even in the outpatient setting, to facilitate diagnosis, ascertain severity of disease, confirming its utility as an excellent biomarker for risk stratification as well as subsequent resource utilization [27]. The NP literature is now extensive and confirms results from previous meta-analysis demonstrating NP's role in diagnosing cardiogenic dyspnea and reducing admission rates [28]. Elevated NPs levels also directly correlate to the respective functional NYHA class, intraventricular pressure, and pulmonary pressure and inversely to cardiac output [18]. 

### 3.2. NP in Risk Stratification

NPs measurements appear to be a useful tool for risk stratification; in fact, high levels are associated with recurrent hospitalization and risk of sudden death. Several studies that used natriuretic peptides in predischarge indicate that BNP levels appear to be the strongest predictor for identifying subsequent death or hospital admission within 6 months [25, 29]. In the Australia-New Zealand Heart Failure Group Trial in patients with chronic HF and reduced systolic function, levels of NT-proBNP above the median were associated with an increased risk for new decompensate HF events and all-cause mortality during the 18-month followup [19]. The largest study is the Valsartan Heart Failure (Val-HeFT) trial in patients with chronic HF who received the recommended medical therapy: an increment of 500 ng/L above the baseline concentration of NT-proBNP carried an increased adjusted risk of 3.8% for mortality and 3.0% for hospitalization for HF. On a multivariate analysis, once again NT-proBNP was ranked as the first prognostic factor in these patients, proving to be independent of and more powerful than traditional risk factors, such as NYHA class, age, left ventricular dilation, or renal dysfunction [30].

### 3.3. NP and Cardiac Performance

NP measurements are also related to several indexes of systolic and diastolic functions. Traditionally, its samples are linearly increased in relation to the degree of systolic dysfunction and cardiac enlargement [20, 31]. Invasive measurements are the reference standard for establishing pulmonary pressure and filling pressure elevation in all subjects with dyspnea, but noninvasive methods of estimating LV filling pressure and pulmonary pressure are entering the current practice. Recently, it has been demonstrated that BNP in systolic HF is progressively increased in relation to the degree of diastolic dysfunction, the severity of mitral valve regurgitation, and right ventricular dysfunction [32]. NPs also reflect elevated LV filling pressure and pulmonary capillary wedge pressure measured invasively with good accuracy and specificity. On the contrary, sensitivity is often modest particularly in patients with preserved systolic function and normal LV volumes [21, 33]. The best correlation between BNP and invasive measurement has been demonstrated with end diastolic wall stress and end diastolic pressure. This suggests that diastolic stretch is one of the major determinants of NP induction [34]. Finally, in a more recent study that compared BNP levels with venous pressure, capillary wedge pressure, and diastolic filling pattern, evaluated by echo, authors showed that as BNP cutoff >400 pg/mL is able to identify patients with higher wedge pressure, it correlates well with all hemodynamic parameters [35].

Some reports have also demonstrated a positive correlation between NP and several markers of LV filling measured traditionally by transmitral Doppler and more recently by tissue Doppler as well as E/E^1^ ratio. However, NP measurements are not able to differentiate between systolic and diastolic dysfunctions [22]. A combined approach with echo Doppler parameters and BNP data seems to be able to stratify patients with systolic dysfunction better. Therefore, tissue Doppler analysis demonstrated that BNP in the gray zone between 8 and 15 is able to differentiate patients with increased filling pressure providing a better stratification. The integrative approach could also improve the diagnostic accuracy in patients with systolic dysfunction and abashed echo diastolic parameters [36]. NP showed a significant elevation together with other parameters of the right ventricular function, increasing with greater dilatation and impairment, as with right and ventricular longitudinal dysfunction. Right ventricular systolic dysfunction is an independent prognostic factor in patients with moderate to severe HF, and it is strictly related to reduced effort tolerance and exercise capacity. In patients with right ventricular pressure overload, NP levels correlated with mean pulmonary artery pressure, right atrial pressure, RV end-diastolic pressure, and total pulmonary resistance [21]. The previously cited measurements have important prognostic power, and the combination of laboratory and imaging data provide a more precise risk prediction for rehospitalization and mortality in patients with HF. 

### 3.4. NP for Therapy Monitoring

Many studies have demonstrated that there is a reduction in NP after the administration of loop diuretics and other drugs during the acute HF phases. This is due to reduced filling pressure and wall stiffness into LV as well as a decrease in idro-saline retention and in neurohormonal overdrive. In a chronic outpatients group STARS-BNP trial, it was clearly shown that a BNP-guided strategy reduces the incidence of death and rehospitalization for HF [23]. More definitive data were reported by Cohen-Solal in acute HF: patients with BNP reduction over 30% after therapy showed a significant reduction in mortality and rehospitalization compared with nonresponders [24]. These results suggest that the variations in BNP concentrations after therapy for acute HF are independent and objective predictors of therapy's adequacy.

For all the previously cited reasons, NP measurement entered in the last HF guidelines providing additive diagnostic and prognostic information with respect to the previous approach (Table 2). 

Such dowries are due to its secretion mechanism: NP, are increased as a consequence of augmented cardiac pressure, volume overload, and wall tension. NP levels are also mediated by means of the renin-angiotensin system and neuroendocrine overdrive; therefore, for specific counterregulatory mechanisms, levels are augmented during idro-saline status retention. Even if NP cannot be considered as “araba fenice,” they should be regarded as the best candidates to provide some additive and complementary information with respect to the traditional tools in the HF setting. 

## 4. Natriuretic Peptides and Diastolic Dysfunction

HFpEF accounts for around 50% of patients with acute decompensated HF. The diagnosis, at times, may be difficult. Three major conditions need to be recognized: (1) signs and symptom of heart failure, (2) preserved systolic function (EF ≥50 %), and (3) left ventricular diastolic dysfunction (increased myocardial stiffness, elevated filling pressures, and abnormal diastolic relaxation) [37]. In clinical practice, differentiating HFpEF from heart failure with reduced ejection fraction (HFrEF) is often difficult on the basis of history, physical examination, chest X-ray, and ECG alone. Assessment of these patients demonstrates a left and upward shift in their end-diastolic pressure volume curve shifted with cardiac chambers dilatation without severe increase of myocardial mass. Invasive measurement is the reference standard for establishing LV end diastolic pressure; however, this is not currently feasible in all subjects with preserved systolic function; therefore, echo-Doppler measuring is the most reliable method of estimating LV pressures [38]. NPs levels are known to be elevated in patients with increased ventricular filling pressures. Pressure overload and ventricular volume expansion are the causes of high levels of this cardiac neurohormone. Hence, NPs can predict diastolic dysfunction increasingly in patients with either symptomatic or asymptomatic diastolic abnormalities. Lubien et al. detected how this peptide can confirm the diagnosis of diastolic heart failure using two-dimensional Doppler echocardiography [39]. The mitral inflow velocity was recorded in most patients; when filling pressures are elevated, E velocity increases, and A velocity decreases producing a restrictive pattern. Tissue Doppler measurements of mitral annular side show a reduced early diastolic velocity (Ea); by this method, the E/Ea ratio can reliably estimate the ventricular filling pressures with reasonable accuracy. If the E/Ea ratio is <8, filling pressure is normal with normal myocardial relaxation, if it is >15 filling pressure is elevated, but if this ratio is between 8 and 15, the assessment of diastolic dysfunction is not clear. In this case, a rapid assay of BNP can detect diastolic dysfunction. Maeder proposed a BNP level > 200 pg/mL to confirm the diagnosis of heart failure with normal ejection fraction in patients with E/Ea in the gray zone [40].

**Figure 2:**
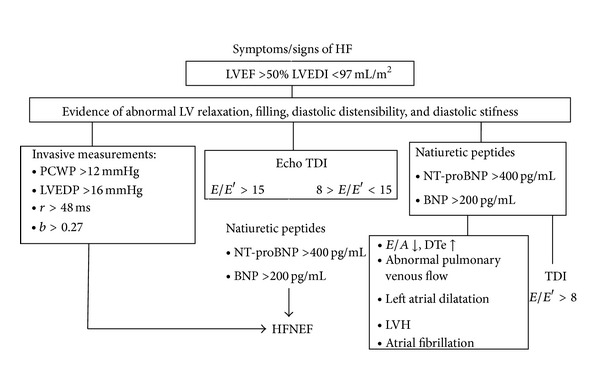
Algorithm including both echo and NP measurements to identify patients with HF and preserved systolic function (modified by Maeder and Kaye [40]).

In patients with HFpEF, BNP increases according to the degree of diastolic dysfunction: the noninvasive analysis of HF with preserved systolic function is often difficult to establish, LV filling estimation appears particularly complicated in those patients with pseudonormal pattern and E/Ea ratio between 8 and 15. In these patients, BNP can detect diastolic filling pressure helping in the screening and grading its severity [41]. These findings have been validated by a comparison between Doppler and invasive hemodynamic measurements, confirming the great reliability of NT-proBNP in diagnosing isolated diastolic dysfunction [42, 43]. In the same setting, NPs are as well related to atrial volume enlargement which is considered an indirect marker of filling pressure even in the absence of primitive mitral valve disease, Figure 2.

## 5. Natriuretic Peptides as a Screening Tool in the General Population

Although NPs have been emerged as a useful tool in the diagnosis of acute HF and are entered also in the American Guideline algorithms, their significance as a screening device for the general population, namely, to detect asymptomatic patients with LV dysfunction, is currently debated [44]. In the general population, NPs measurements are affected by several cardiac and noncardiac variables (see the following paragraph) that need to be taken into account during the patient's evaluation. Before the clinical syndromes of HF are displayed, several haemodynamic and LV pressure-volume modifications occur: the first step is an abnormal systolic or diastolic function that is succeeded by increased end diastolic pressure and wall tension. After this stage, the LV pressure-volume curve shifts downward to the right; in the remodelled heart, there is a further increase in LV filling, impaired relaxation with stiffness, and increased left atrial pressure. Clinical syndromes of HF with symptoms of fatigue, dyspnea, dizziness and so forth, begin to be evident at this stage, and it will be clearly evident when increased left chamber pressure reflects a raise in pulmonary pressures [45]. Importantly, these clinical syndromes represent the tip of the iceberg, as it would be ideal to recognize cardiac remodelling before the clinical syndrome is apparent during early stages (stages A and B) of the last HF classification. The importance to intercept patients during these stages is confirmed by epidemiological data showing that asymptomatic patients with mild to moderate LV dysfunction have worse outcomes. Whether NPs screening would recognize this process before clinical manifestations become evident is debated. Some reports seem to confirm a role in this context; in Olmstead county a study on 2042 subjects NTpro-BNP and BNP demonstrated high sensitivity and specificity in detecting moderate systolic and diastolic dysfunctions (86 and 81%, resp.) particularly in older patients [46]. In another report with a 3-year median followup, NT-proBNP was the strongest predictor of mortality and hospital admission in asymptomatic patients with evident cardiac dysfunction. Its values increased with the severity of cardiac dysfunction [47]. Opposite data were reported from Framingham in detecting elevated LV mass and systolic dysfunction. However, this study included both patients at a high risk and after myocardial infarction, including a wide range of values [48]. More recently, Costello-Boerrigter et al. found that NT-proBNP is an effective tool to make out patients with systolic dysfunction, while the identification of diastolic dysfunction was less effective [49]. However, the combination of BNP with Tissue Doppler seems to be able to recognize community patients with an increased risk and diastolic impairment [50]. The latter data supports the double echo and laboratory approaches in the detection of both systolic and diastolic dysfunctions, confirming NP's role as potential predictive marker of adverse outcome even in asymptomatic patients. Nevertheless, at this moment, a common cutoff does not exist, neither for BNP nor for NT pro-BNP that is universally accepted and clinically applicable as a screening tool in general population.

## 6. Limitations of Natriuretic Peptides 

Looking all together at the previously cited data, NPs measurement could appear to be the best solution for few diagnostic and prognostic troubles in clinical practice: measuring NPs, we are able in theory to obtain a variety of information on HF diagnosis, severity, and identification of patients with poor outcome. Unfortunately, several conditions can potentially influence NPs measurements, as demonstrated by Framingham and the Dallas Heart Study data in which a BNP cutoff >80 was able to identify only subjects with severe systolic dysfunction in the general population [48, 51].

Physiologic status race, sex, age, and body mass index are all conditions that could alter NP's synthesis and clearance [52]. Redfield et al. confirmed the impact of age and sex on BNP observed in subjects without cardiovascular disease; NPs measurements would be used taking into account discriminatory values adjusted for sex and age [53]. It has been demonstrated that age increases the levels of circulating BNP; this is related to a decline in myocardial function and myocardial fibrosis cardiovascular stiffness and the reduction in clearance of natriuretic peptides typical for senescence. However, an exact cut-off value for the difference among sample methods as for a wide variability is not well established in the literature. NPs are also inversely related to body mass, and patients with higher body mass index revealed lower circulating BNP concentrations [54]. This inverse relation may be due to increased expression of NP clearance receptors by adipose tissue, resulting in an increased clearance of NP in obese subjects. Race is another factor that could influence plasma NP levels: Hispanic and Black races have higher levels with respect to the Caucasian population for each corresponding NYHA class [55]. Besides physiologic conditions, several inflammatory and systemic diseases can affect NP values [56]. 

### 6.1. Comorbidities

The most important conditions are comorbidities like renal insufficiency (RI), diabetes, and anemia that are often associated with chronic HF particularly in older patients with more advanced stages [57]. Many studies demonstrated that the impact of RI on NPs is independent from the cardiac function. It depends not only on the RI severity but also on the duration of disease [58].

Influential authors calculated that, in the presence of these associated conditions, NPs values are at least 1/3 higher than those in patients with normal renal function [59]. Anemia is another condition frequently associated with more advanced HF stages, and its correction could reduce NPs levels [60, 61]. 

 Cut-off limits of NPs have a grey zone in which it is not possible to ascertain their exact diagnostic role, their accuracy, and their predictive values [62]. This range is between 100 and 400 for BNP and 400 and 1400 for NT-pro-BNP; when values fall into these intermediate concentrations, it is not possible to have enough accuracy, and further clinical and investigational analyses need to be performed to make a diagnosis in patients with acute dyspnea. Furthermore, the outcome of patients who fall into this intermediate range is not clear [63, 64]. Another limitation in clinical practice is the moment of the measurement: NPs are released by increased volume load and wall stretch, which are influenced by systemic hydrosaline retention (wet versus dry). After treatment with drugs of proven efficacy, we observed a progressive reduction that is considered as a “measure” of congestion. Their prognostic role also depends on the time of measurement that reflects the volume status.

Finally, several cardiac factors can cause NPs alterations even in the absence of increased filling pressure and frank HF: increased LV mass, reduced right ventricular function, mitral valve disease, high pulmonary pressure for primitive or secondary respiratory disease, pulmonary embolism, and atrial fibrillation are all causes of potential NP increase. Overall, these reasons for NP measurement have high sensivity but low specificity, and laboratory test needs to be confirmed by clinical and traditional diagnostic screening processes. 

## 7. Conclusions

NPs are important biomarkers able to assess diagnosis and severity of heart failure as well as predict outcome and potentially guide therapy. Although echocardiography remains the standard regarding detailed information on cardiac performance and structure, imaging data alone is not sufficient to better identify patients with adverse outcome. An integrated approach combining laboratory assays with imaging could lead to a better identification of patients at high risk. This behaviour seems particularly useful in some settings like in patients with isolated diastolic dysfunction as in asymptomatic patients. In patients with diastolic dysfunction, it is sometimes difficult to confirm diagnosis, and NP should help to identify and graduate diastolic dysfunction. In community patients, NPs diagnostic, and prognostic values need to be validated although it is an effective tool to recognize patients with moderate to severe systolic dysfunction. The major limitation consists in the lack of a universally accepted cutoff that could permit the application of NP assays to clinical practice in this setting. The hope is that in the future multimarker strategies together with specificity and cut-off improvements could accurately and early identify patients “under the iceberg” that will develop HF syndrome. In this ideal world biomarkers on one hand and imaging on the other hand could converge to identify patients with higher risk. To obtain this, we need to better standardize echo as well as laboratory parameters. 

## Figures and Tables

**Table 1:** Classification of biomarkers for early cardiac damage identification.

Markers of neurohormonal activation	(i) Natriuretic peptides (ANP, BNP, CNP, and related peptides) (ii) Markers of renin angiotensin aldosterone system activity (iii) Arginine vasopressin

Markers of cardiac injury	(i) Cardiac troponins (cTnI, cTnT) (ii) Heart-type fatty-acid protein (iii) Myosin light-chain kinase I

Markers of inflammation and matrix remodelling	(i) Matrix metalloproteinases (MMPs) and MMPs tissue inhibitors (ii) C-reactive protein (iii) Cytokines and related receptors (interleukins IL-1, IL-2, IL-6, IL-8, and IL-18, TNF-*α*, osteoprotegerin, and Fas ligand)

Markers of systemic organ damage	(i) Renal injury: creatinine, BUN, NGAL, and cystatin C (ii) Hepatic injury: transaminases and gamma-glutamyl transferase

Aspecific laboratories indexes	(i) Anemic status and erythropoietin endogenous levels (ii) Low albumin levels (iii) Hyponatremia (iv) Carbohydrate antigen 125

B-type natriuretic peptide (BNP), atrial natriuretic peptide (ANP), C-type natriuretic peptide, tumor necrosis factor-*α* (TNF-*α*), blood urea nitrogen, neutrophil gelatinase-associated lipocalin (NGAL).

**Table 2:** Clinical trials regarding NPs measurement for diagnosis, cardiac dysfunction, and therapy monitoring.

Author	Clinical setting	Patients	Study design	Main findings
Maisel et al. [16]	Patients with acute decompensated heart failure (ADHF).	464	Entrance criteria included a BNP level >100 pg/mL. Admitted patients were divided into two groups based on B-type natriuretic peptide (BNP) levels above and below 200 pg/mL to study differences in outcome rates.	The BNP levels can predict future outcomes and thus may aid physicians in making triage decisions about whether to admit or discharge patients. Emerging clinical data will help further refine biomarker-guided outpatient therapeutic and monitoring strategies involving BNP.

Luchner et al. [17]	Patients with ADHF	1086	Primary endpoint was hospital admission; secondary endpoints were intermediate/intensive care unit (IMC/ICU) admission, length of stay, rehospitalization and death, or rehospitalization.	Knowledge of N-terminal pro-B-type natriuretic peptides (NT-proBNP) had no significant effect on the primary endpoint hospital admission and the secondary endpoints. Patients with high open NT-proBNP (>1800 pg/mL) were more likely to be admitted to the hospital and IMC/ICU, whereas patients with low open NT-proBNP (<150 pg/mL) were less likely to be admitted compared with patients with blinded NT-proBNP.

Bettencourt et al. [18]	Patients with ADHF	182	The goal of the study was to evaluate the value NT-proBNP in predicting death or hospital readmission after discharge of HF patients.	Variations in NT-proBNP levels are related to hospital readmission and death within 6 months. NT-proBNP levels are potentially useful in the evaluation of treatment efficacy and might help clinicians in planning discharge of HF patients.

Masson et al. [19]	Patients with chronic and stable HFs.	3916	This work aimed to provide a direct comparison of the prognostic value of BNP and NT-proBNP in patients with chronic and stable HFs.	BNP and NT-proBNP showed subtle differences in their relation to clinical characteristics and prognostic performance in a large population of patients with chronic and stable HF. They were the most powerful independent markers of outcome in HFs.

Troughton et al. [20, 21]	Patients with systolic heart failure (SHF).	106	This study was designed to characterize the importance of echocardiographic indexes as determinants of BNP levels in patients with SHF.	Plasma BNP levels are significantly related to diastolic indexes measured from tissue Doppler imaging (TDI) and color M-Mode in SHF. BNP levels reflect the severity of diastolic abnormality, right ventricle (RV) dysfunction, and mitral regurgitation. These findings may explain the powerful relationship of BNP to symptoms and prognosis in SHF.

Bistola et al. [22]	Patients with advanced chronic HF (CHF).	102	Patients with CHF were studied by 2-dimensional conventional and TDI echocardiographies of the left and right ventricles. Patients were followed for 6 months for major cardiovascular events.	RV TDI systolic velocity, dilated cardiomyopathy, digoxin treatment, and female gender were associated with increased cardiovascular death. RV TDI indexes combined with increased plasma BNP additively predict adverse cardiac outcomes in advanced CHF.

Jourdain et al. [23]	New York Heart Association functional class II to III patients with CHF	220	Patients with CHF considered optimally treated were randomized to medical treatment according to either current guidelines or a goal of decreasing BNP plasma levels <100 pg/mL. The primary combined endpoint was CHF-related death or hospital stay for CHF.	In optimally treated CHF patients, a BNP-guided strategy reduced the risk of CHF-related death or hospital stay for CHF. The result was mainly obtained through an increase in ACEI and beta-blocker dosages.

Cohen-Solal et al. [24]	Patients with ADHF	1327	The purpose of this analysis was to examine whether decreases in BNP levels during the first few days of hospitalization were associated with greater survival in patients with ADHF.	Patients with lowered BNP on treatment for ADHF had reduced mortality risks (31- and 180-day) compared to those with little or no BNP decrease. These results suggest that early lowering of BNP predicts both short- and long-term mortality risks. BNP reduction may therefore serve as a suitable prognostic marker of all cause mortality.

Richards et al. [25]	Patients with ischemic LV dysfunction	297	They sought to assess plasma concentrations of NT-proBNP and adrenomedullin for the prediction of adverse outcomes and responses to treatment.	In patients with established ischemic LV dysfunction, plasma concentrations of NT-proBNP and adrenomedullin are independent predictors of mortality and HF. Carvedilol reduced mortality and HF in patients with higher pretreatment plasma NT-proBNP and adrenomedullin.
